# Dr. Osborn's Sketch of the Physiology and Pathology of the Urine

**Published:** 1821-06-01

**Authors:** 


### *A Sketch of the Physiology and Pathology of Urine; with an Historical Introduction.* By JONATHAN OSBORNE, M.B.T.C.D. Licentiate of the King's and Queen's College of Physicians in Ireland.

THE phenomena, which the urine presents, attracted considerable attention even in the earliest ages, as may be seen in the writings of Hippocrates and of other ancient authors. From some cause, probably from the prevalence of uromancy and an enactment of the London College, to suppress it among physicians, the study of the excretions was for a long period made the subject of ridicule. In this age of science, however, it was natural to expect uriology would be revived, particularly as the discoveries in animo-chemical philosophy have rendered the pursuit comparatively easy. The different analyses of the urine lie scattered in various publications, and it has been the aim of the author of the Treatise before us to collect and arrange them, without making any pretentions to originality.

"Thus the following Treatise may be regarded as an index of what has been hitherto discovered, concerning the urinary secretion, and is the result of an attempt to frame for any person interested in the subject, such a sketch as I should have been desirous to possess, when I commenced my inquiries." *Pref. p.* 6.

The colouring matter of urine is soluble in water, and when an extract of urine has been obtained by evaporation, it gradually concretes into a mass of crystals. All that is soluble in alcohol may be separated from the rest by macerating the mass with four times its weight in that fluid for some time. The decanted liquor, having been slowly evaporated to the consistence of syrup, presents, on cooling, the substance called *urea* in tabular, quadrangular crystals. This may also be detected by nitric acid. Urea is stated by Berzelius* to be a compound mass.

Uric acid is precipitated spontaneously and gradually acquires a reddish hue. It may also be diluted by nitric acid.

The lactic, the phosphoric, the muriatic, and sulphuric acids, may also be detected in the urine; and carbonic acid, which was long suspected to exist in it, was discovered by

* Berzelius' View of the Progress and present State of Animal Chemistry being out of print, we think that a more acceptable present could not be made to the Profession, than a second edition. The rapid sale of the work is a proof of the avidity with which the subject is pursued. *Rev.*

Vogel. The presence of carbonic acid is, however, by no means constant. Fluoric acid has been found by Berzelius.

Potash, soda, lime, magnesia, and ammonia, have been proved to exist in urine, and by various processes sulphur and silex.

The mucus of the bladder is separated by filtration.

The urea, uric acid, mucus, and the earthy phosphates, are constantly changing their proportions.

Healthy urine is always acid, and remains so for a few days, and in winter it has been found to redden litmus-paper nearly three weeks. Berzelius thinks this acidity is derived from the lactic, uric, and carbonic acids.

The specific gravity of urine ranges from 1010 to 1020.

The quantity and qualities of the urine are influenced by cutaneous exhalation and other evacuations, by exercise, by diuretics, age, posture, abstinence, various kinds of diet and medicine, particularly by acids and alcalies.

In the analysis of the urine, that passed in the morning, is always to be preferred, and it should, previously to an examination, be suffered to cool. It is sometimes turbid, as soon as it is voided. This arises from an excess of alcali, or of the earthy phosphates, or from the admixture of mucus or of blood. Exposed to 160 degrees of heat it coagulates, when much albumen is present; and when this test fails, albumen may be discovered by nitric acid, or, with certain precautions, by the oxymuriate of mercury.

In examining the urine in febrile diseases, we observe the following phenomena: a greater specific gravity and higher colour than in health; the mucous cloud floats towards the surface, or is absent; a precipitate is formed with the oxymuriate of mercury; there is a tendency to become scanty. Fever is sometimes accompanied with *ardor urinæ* and *dysury*, occasioned by the deficient secretion of mucus in the bladder and urethra. These symptoms are particularly evident in hepatic affections, as have been noticed by modern observers.*

In opposition to the opinion of Dr. Scudamore, the author believes that the precipitation caused by the oxymuriate of mercury is a characteristic property of inflammatory urine, and that it appears to have some analogy with the buffy coat of the blood. The diseases furnishing most of his observations were continued fevers, hepatitis, phthisis pulmonalis, nephritis, rheumatism, and pneumonia.

---

* See an Essay on the Influence of Tropical Climates. By Dr. James Johnson, 8vo. London, 1818.

The crisis of continued fevers is commonly announced by a copious lateritious, or pink-coloured sediment. The same appearance takes place in the urine during the course of other diseases, as gout, acute rheumatism, and some chronic disorders, the exact nature and cause of which we have not been able to ascertain. Dr. Scudamore is of opinion that the appearance of these sediments is entirely dependent on a faulty state of the digestive organs, and on unhealthy assimilation; and we agree with him in believing, that in these cases not only are the digestive functions irregular, but that the different secreting organs are much influenced by a nervous state of the constitution.

In putrid or malignant fevers the urine is alcaline, when passed, and contains a great quantity of free ammonia and less urea than healthy urine; and in the plague and small pox, bloody urine is esteemed an almost fatal sign.

When the viscera of the thorax are inflamed, the urine is almost invariably high coloured. It is also high-coloured in hectic fever, and continues to deposit a lateritious sediment without any abatement of the febrile symptoms.

Mr. Rose found the urine in hepatitis to be void of urea, and Dr. Henry that in chronic hepatitis to be free from colour and odour. In the latter instance, as the patient recovered, the urea was gradually restored. Dr. Osborne's experience does not confirm these observations.

"In the hepatic patients, which" (whom) "I have seen, the urine had a high colour, proportionate to the degree of fever which existed; and I rather suspect that the urine described by Dr. Henry, must have been occasioned by the inflammation having extended to the kidneys. However, this subject must remain undecided, till more numerous and decisive observations are obtained." P. 60.

Glands in a state of inflammation cannot well perform their functions: hence the urine, discharged during nephritis, is described by some of the systematic authors to be scanty and pale. Most frequently it appears red, and more or less turbid, from small quantities of blood emitted by the renal capillaries; or from a coagulum formed in the bladder, which occasions remarkable appearances, described and explained by Sir Everard Home, in the Philosophical Transactions for 1797. When suppuration ensues, there is a purulent discharge with the urine, which is turbid, emits a peculiar fœtor, and deposits a milky sediment; and in gangrene, the evacuation has been observed high-coloured and turbid. The different terminations of nephritis, however, cannot be determined entirely by the discharge from the urinary organs.

By numerous experiments it has been proved that, during a fit of the gout, the urine contains an increased quantity of phosphoric acid; its specific gravity is augmented, ranging from 1025 to 1040; and in the urea there is a proportional increment. The fluid is less abundant, and of a deeper colour than usual; and on cooling, deposits the pink or lateritious sediment.

An examination of the urine in dropsical diseases is of the highest importance. Mr. Cruickshank appears to have been the first, who noticed its coagulation on the application of heat or nitric acid. Dr. Rollo also found that the urine in acute rheumatism is coagulable. This subject has of late received particular attention from Dr. Blackhall, who observes, that coagulable urine attends universal dropsy, and is often superadded to great visceral unsoundness. He states that when the urine is most loaded, it coagulates by the lowest heat, and with the greatest firmness; while the blood is always found the most buffy, and the system exhibits the strongest marks of inflammation. Dr. Osborne thinks that Dr. Blackhall's enumeration of cases of uncoagulable urine affords no positive information, as the urine appears not to have been tried by nitric acid as well as by heat. Dr. Wells attributes the failure of the latter test, when nitric acid afterwards succeeds in producing coagulation, to a deficiency of salts in the urine. Any neutral salts being added to serum coagulable only by nitric acid, a coagulation will be produced on the application of heat. Dr. Wells' analysis of dropsical urine shows that diffused dropsy, when not preceded by some debilitating disease, is generally accompanied by coagulable urine; and that encysted dropsy and ascites are most frequently not so accompanied; and it also tends to confirm the assertion of Cruickshank, that in the dropsy proceeding from unsound viscera, the urine is usually uncoagulable. M. Nysten remarks that, in general, when dropsical patients pass as much urine as equals in quantity that of their beverage, and when diuretics begin to take effect, the fluid discharged differs very little from natural urine. In the commencement of hydrocephalus some have observed milky urine, and it is stated by Dr. Coindet, that in the second stage a micacious deposition occurs, resembling the crystals of boracic acid, which he believes to be urea, and regards as an appearance peculiar to the disease.

The saccharine matter, obtained from the urine in paruria mellita (diabetes) is discovered not only by its sensible qualities, but by the effects produced on it by nitric acid, and by the production of alcohol from it, in almost the same proportion as from sugar by means of fermentation and distillation. Its specific gravity ranges from 1028 to 1040, and in one patient, who died, we found it to be 1045. The formation of sugar nearly supersedes that of urea; but the other salts exist in the same proportion to each other as in health. In one of our patients, who died of what Dr. Rollo denominated cœliac diabetes, at the beginning of his disease, when the blood was highly inflammatory, and only two scruples of saccharine matter were produced by evaporating four ounces of his urine, coagulation took place; but when we endeavoured towards the decline of the disease to obtain this result, we could not succeed. Dr. Bostock analyzed the urine in one case of diabetes insipidus, and obtained no sugar; but the urea and the salts of urine were in such excess, that six ounces of urea, and above an ounce and a half of salts more than the natural quantity, must have been discharged daily.

Nervous urine, such as is voided by hysterical and hypochrondriac patients, loses most of the colouring matter, contains more urea and uric acid, than urine emitted soon after drink, but not so much as digested urine. Similar urine is passed in spasmodic diseases.

The urine is of a yellow or greenish yellow colour in jaundice, and has been proved by Orfila and others to contain bile. Muriatic acid is a good test of the presence of bile in the urine: in a short time a small quantity produces with it a green colour.

The urine of children afflicted with worms is generally observed to become milky soon after it has been voided. According to Fourcroy, its sediment exhibits all the properties of oxalate of lime.

In rickety children the urine has been proved to contain much phosphate of lime, although it was found to be deficient in the bones of those, whose urine was examined. Both worms and rickets we suspect are preceded and accompanied by functional disorders in the chylopoietic viscera, which has the effect of suspending the process of ossification.* That disease of structure is not essential to interrupt the formation or deposition of phosphate of lime is probable; because in one rickety child we found all the abdominal viscera perfectly sound, the glands of the mesentery in particular; and in another, whose bony fabric was unaffected by disease, the whole of the abdominal viscera, excepting

---

* See a Practical Treatise on the Remittent Fever of Infants, &c. By J. M. Coley. Underwood and Co. London, 1813. Page 153.

the alimentary canal and the urinary organs, were obliterated by the tubercular disease described by Dr. Baron. Not a vestige of the liver, pancreas, or spleen, was to be found.

An oily film is to be seen on the urine in various dyspeptic complaints, which Dr. Osborne thinks appears to be derived from a depraved secretion of mucus, and deposits an abundance of the earthy phosphates. These appearances usually cease after the use of purgatives, and seem to be closely connected with the state of the bowels. Those who lead a sedentary or inactive life, in universities and similar situations, are most subject to the disease. Considerable irritation is apt to be felt at the neck of the bladder, attended with a frequent desire to make water, which are both relieved by muriatic acid.

An excellent and useful table of urinary calculi, representing their sensible and chemical properties, comparative frequency, &c. conclude the pamphlet.

Notwithstanding we have been presented with many chemical and pathological researches concerning the urine, the science of uriology is still in its infancy. If Dr. Osborne has not made any discovery in this department, he has facilitated the study of it, and rendered it more inviting; and while we commend him for laying a foundation with materials ready prepared by others, we hope to see the structure enriched by his own workmanship.

Having often observed the symptoms of gravel attended with a discharge of crystals of uric acid, after the potation of stale beer, which did not impart any sensible acidity to the taste, we were induced, while reading Dr. Osborne's work, to make a few experiments with the view of ascertaining the cause of the formation of this acid in excess. To twelve ounces of healthy urine we added three of stale beer, of the kind above stated. In twenty-four and forty-eight hours no change had taken place; but in seventy-two hours a small quantity of very minute crystals of uric acid were deposited at the bottom of the vessel. Carbonate of soda being added to another portion of the beer, small bubbles of carbonic acid were seen slowly to ascend. From these experiments it is evident that the acetic acid in the beer has the effect of disengaging the uric acid from the substance with which it was united, by the power of a superior affinity; but we apprehend that this process does not take place in those persons from drinking stale beer, whose digestive powers are strong.

